# Innovative ultrasound-guided one-stop surgery for complex cardiovascular cases: a case report

**DOI:** 10.1093/jscr/rjad696

**Published:** 2024-01-04

**Authors:** Meng Zhang, Xinyi Ma, YanHui Li

**Affiliations:** Department of Cardiology and Echocardiography, the First Hospital of Jilin University, No. 71 Xinmin Street, Changchun, 130021, China; Department of Cardiology and Echocardiography, the First Hospital of Jilin University, No. 71 Xinmin Street, Changchun, 130021, China; Department of Cardiology and Echocardiography, the First Hospital of Jilin University, No. 71 Xinmin Street, Changchun, 130021, China

**Keywords:** one-stop surgery, operative transcatheter aortic valve implantation, heart septal defects, atrial echocardiography, transesophageal imaging

## Abstract

Elderly patients with multiple comorbidities often face complex cardiac challenges, including aortic valve issues and atrial septal defects. Traditional open-heart surgery may not be viable for this demographic. Transcatheter aortic valve implantation (TAVI) emerges as a preferred alternative. In this case, a frail patient with multiple comorbidities, atrial septal defect, and significant aortic stenosis and regurgitation underwent a one-stop procedure, combining TAVI and atrial septal defect closure, guided by advanced imaging, including three-dimensional ultrasound. Ultrasound played a pivotal role in the perioperative phase, offering precise screening and guidance. This innovative technique, minimizing surgical trauma and recovery time, significantly improved the patient’s quality of life.

## Introduction

Elderly patients with complex cardiac conditions face challenges due to the risks of conventional open-heart surgery [1, 2]. Transcatheter aortic valve implantation (TAVI) is becoming the preferred alternative for high-risk patients, particularly those with multiple health issues [3, 4]. This article highlights a case of an elderly patient with comorbidities, atrial septal defects, aortic stenosis, and regurgitation. The study focuses on integrating TAVI and atrial septal defect closure in a one-stop procedure, utilizing advanced imaging and a skilled medical team for its success.

## Case report

A 73-year-old male patient presented with exertional chest tightness and shortness of breath 6 months ago. Subsequently, these symptoms recurred and gradually worsened. One month ago, the patient’s condition significantly deteriorated, and transthoracic echocardiography (TTE) diagnosed him with degenerative aortic valve stenosis, moderate to severe aortic valve narrowing with moderate regurgitation, and a secondary atrial septal defect. Given the patient’s advanced age and multiple comorbidities, open-heart surgery was deemed high-risk, and a ‘one-stop’ approach was planned, involving TAVI and atrial septal defect (ASD) closure.

**Figure 1:**
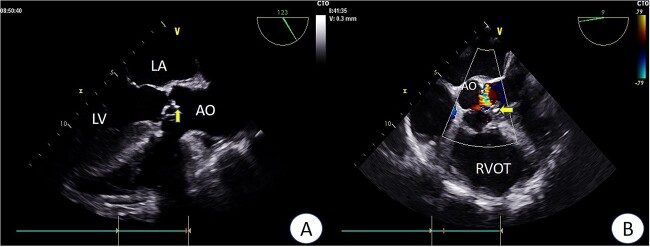
Preoperative TEE: Aortic valve leaflets, calcification, and regurgitation observed in the short-axis section of preoperative TEE. Key abbreviations: LA - left atrium; LV - left ventricle; AO - aorta; RVOT - right ventricular outflow tract.

**Figure 2:**
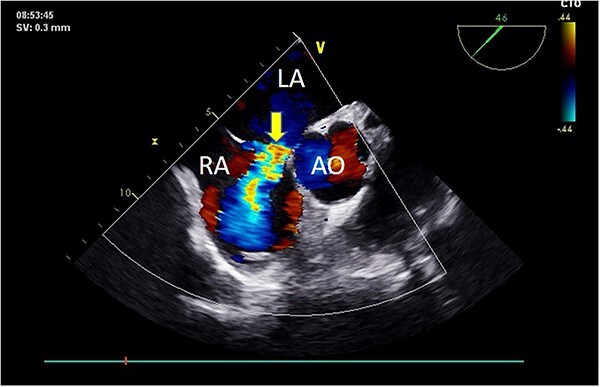
Preoperative TEE: Short-axis view reveals left-to-right atrial septal shunt with a 9 mm blood flow beam (indicated by arrow). Abbreviations: LA - left atrium; LV - left ventricle; AO - aorta.

Preoperative TEE revealed moderate calcification, leaflet thickening, and hypertrophy of the tricuspid aortic valve (Fig. 1). The valve leaflets were restricted in motion, with a calculated maximum valve area of ~1.1 cm^2^ using the continuity equation. Color Doppler flow imaging demonstrated moderate aortic regurgitation. Additionally, a 9 mm-wide ASD was found in the mid-septal region (Fig. 2), with residual lengths on either side. Color Doppler revealed left-to-right shunting at the atrial level.

**Figure 3:**
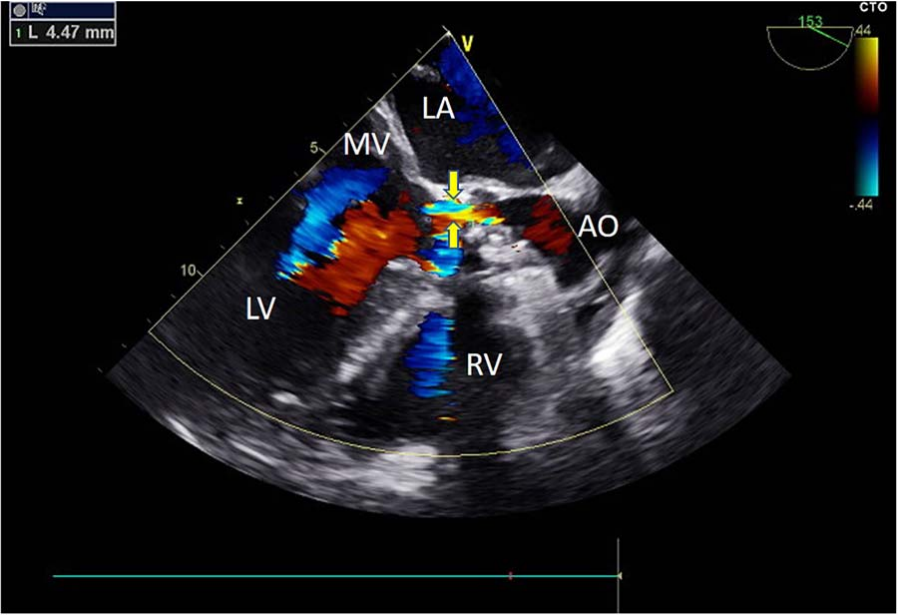
Intraoperative TEE: Long-axis aortic view displays a 4.5 mm-wide perivalvular blood flow bundle around the aortic valve (arrow). Abbreviations: LA - left atrium; LV - left ventricle; RV - right ventricle; AO - aortic.

**Figure 4:**
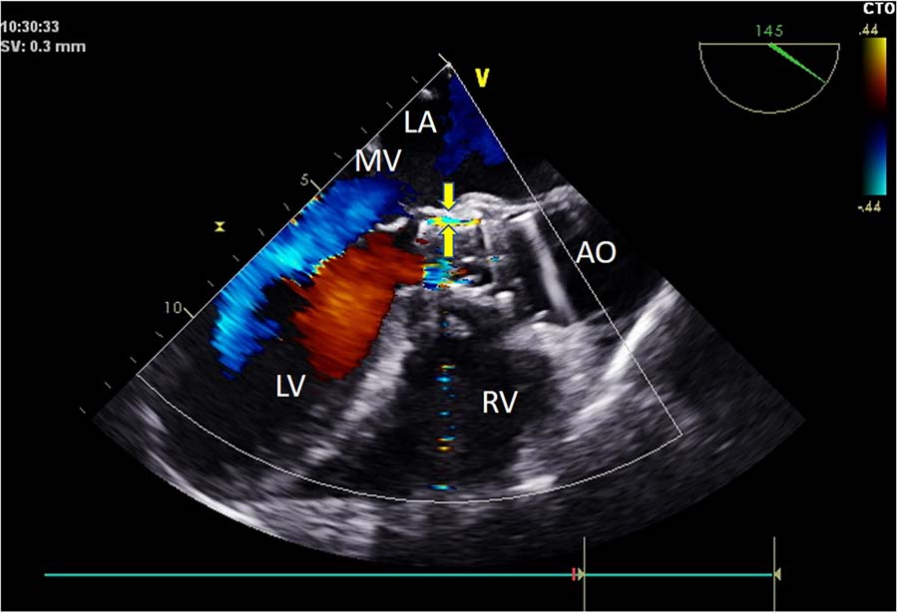
Intraoperative TEE: Long-axis aorta section shows a 1.5 mm-wide paravalvular blood flow beam, demonstrating significant improvement. Abbreviations: LA - left atrium; LV - left ventricle; RV - right ventricle; AO - aorta; MV - mitral valve.

**Figure 5:**
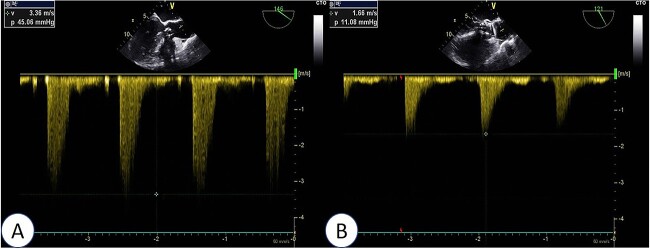
Intraoperative TEE: Post-release of self-expanding valve, aortic valve’s maximum flow velocity measures 166 cm/s, with a pressure difference of 11 mmHg (B), a significant improvement compared to pre-surgery (A: maximum flow velocity of 336 cm/s, pressure difference of 45 mmHg).

**Figure 6:**
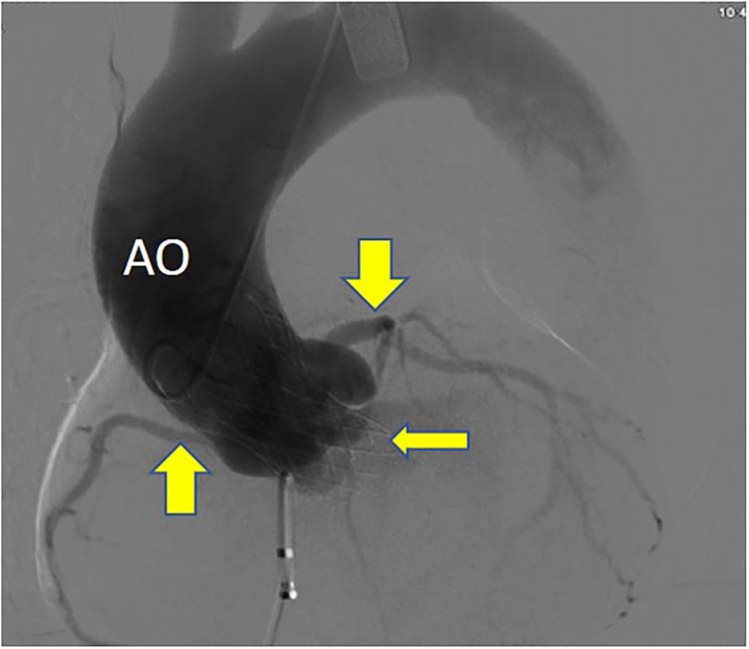
Postoperative DSA: Self-expanding valve holder’s shape and position (thin arrow) are normal, while left and right coronary artery openings (thick arrow) are unobstructed with clear blood flow.

Intraoperatively, after self-expanding the aortic valve, TEE monitoring confirmed excellent cardiac function, disappearance of aortic valve regurgitation, secure anchoring of the aortic valve prosthesis, and no obstruction to the mitral valve leaflets or coronary ostia. The aortic valve velocity and pressure gradient upstream of the valve decreased compared to preoperative values, with a maximum velocity of 166 cm/s and a pressure gradient of 11 mmHg (Fig. 3). However, a periprosthetic leak with a residual width of ~4.5 mm was observed at the self-expanding valve’s interface with the native annulus (Fig. 4). Based on preoperative data, the decision was made to re-expand and reshape the prosthesis. Postdilation, TEE monitoring showed a reduction in the periprosthetic leak to 1.5 mm (Fig. 5), a significant improvement compared to predilation. Following satisfactory self-expansion results, the planned percutaneous ASD closure procedure was performed under ultrasound guidance. The closure device was securely positioned in the atrial septum, and TEE examination showed no deformation or impingement of the self-expanding valve (Fig. 6). Atrial shunting was eliminated, and the outcome exceeded expectations.

## Results

**Figure 7:**
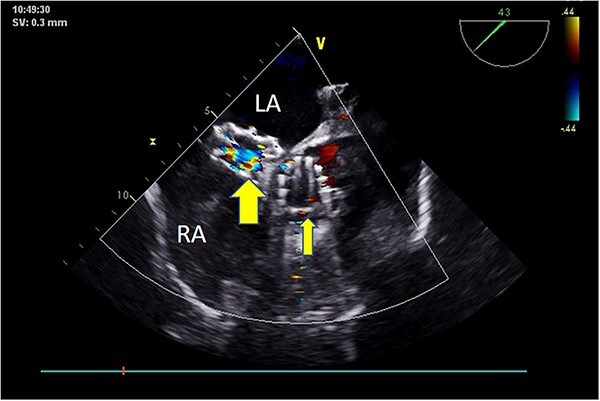
Postoperative TEE: Short-axis TEE displays circular echoes in the self-expanding valve (thick arrow) with good morphology and position. The sealing umbrella (thin arrow) maintains a proper shape and fixed position, eliminating left-to-right atrial-level shunt.

**Figure 8:**
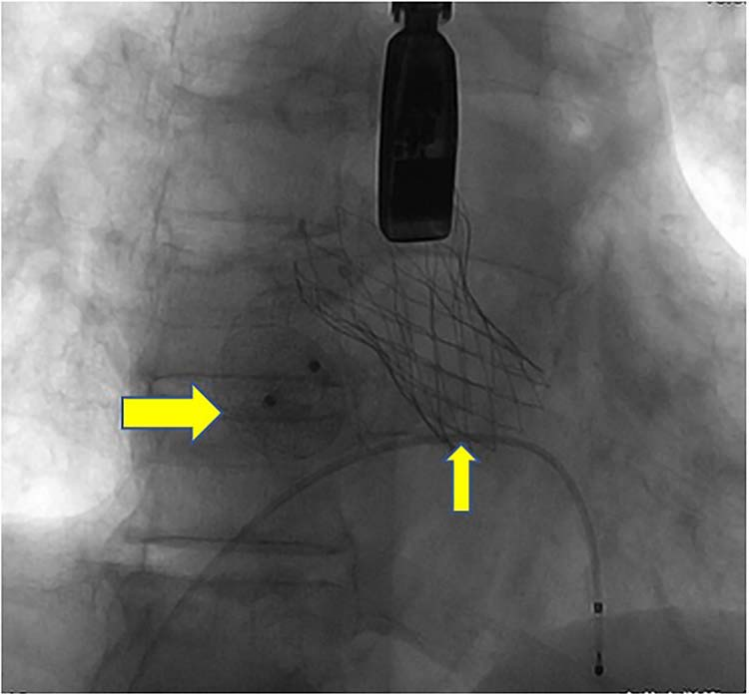
Postoperative DSA: Digital subtraction angiography confirms the proper positioning of the aortic self-expanding valve (thin arrow) and occluder (thick arrow).

Postoperatively, imaging studies, including TEE and digital subtraction angiography (DSA), confirmed normalization of aortic valve flow velocity and pressure gradients, with the absence of aortic regurgitation (Fig. 7). Atrial shunting disappeared, and there was no involvement of the mitral or tricuspid valves. Both the self-expanding valve prosthesis and the closure device were stably positioned, and the surgical outcome was deemed successful (Fig. 8). One month after surgery, the patient’s symptoms of chest tightness and shortness of breath had resolved.

## Discussion

The patient in this case presented a complex medical challenge due to advanced age, frailty, and various health issues. Additionally, the patient had aortic valve stenosis, regurgitation, and an atrial septal defect, which severely affected their heart function. Traditional open-heart surgery was too risky. After extensive consultation with a multidisciplinary team, including cardiac surgeons and specialists, a one-stop procedure combining aortic valve replacement and atrial septal defect closure was chosen. This innovative approach not only addressed the patient’s complex heart issues but also significantly reduced the risks associated with open-heart surgery. The patient’s rapid recovery and discharge within 5 days demonstrate the success of this groundbreaking procedure in cardiovascular surgery.

Preoperative evaluation played a crucial role in ensuring the success of this one-stop procedure. The comprehensive assessment of cardiac function, facilitated by both TTE and TEE, allowed for a detailed understanding of the extent of stenosis above and below the aortic valve [3, 5]. Furthermore, TEE provided real-time guidance during the procedure, enabling precise deployment of the self-expanding flap and necessary surgical adjustments [6]. Postoperatively, TTE continued to be invaluable, monitoring the patient’s recovery and confirming the procedure’s success without inducing adverse reactions.

The significance of ultrasound in this context cannot be overstated. It played a pivotal role in the perioperative phase, serving as a cornerstone for patient screening, the quantification of aortic valve stenosis, selection of appropriate valve sizes, and the determination of optimal surgical approach sites. Its essential role extended to the immediate postimplantation phase, contributing to a comprehensive evaluation of cardiac function, especially in localizing and quantifying paravalvular leaks and valvular regurgitation.

## Conclusion

In conclusion, this case exemplifies the successful application of an innovative one-stop surgical approach in a high-risk patient with complex cardiovascular conditions. The careful preoperative evaluation, real-time intraoperative guidance, and robust postoperative monitoring, all facilitated by advanced ultrasound technologies, were instrumental in achieving a favorable outcome.

## Data Availability

We respect the patient’s right to privacy and to protect her identity, we do not wish to share our patient’s data. We have presented all the necessary information about the case report.

## Statement

The manuscript has been read and approved by all the authors, that the requirements for authorship as stated earlier in this document have been met, and that each author believes that the manuscript represents honest work.
